# Age and Sex Composition of Seals Killed by Polar Bears in the Eastern Beaufort Sea

**DOI:** 10.1371/journal.pone.0041429

**Published:** 2012-07-19

**Authors:** Nicholas W. Pilfold, Andrew E. Derocher, Ian Stirling, Evan Richardson, Dennis Andriashek

**Affiliations:** 1 Department of Biological Sciences, University of Alberta, Edmonton, Alberta, Canada; 2 Wildlife Research Division, Science and Technology Branch, Environment Canada, Edmonton, Alberta, Canada; Phillip Island Nature Parks, Australia

## Abstract

**Background:**

Polar bears (*Ursus maritimus*) of the Beaufort Sea enter hyperphagia in spring and gain fat reserves to survive periods of low prey availability. We collected information on seals killed by polar bears (*n* = 650) and hunting attempts on ringed seal (*Pusa hispida*) lairs (*n* = 1396) observed from a helicopter during polar bear mark-recapture studies in the eastern Beaufort Sea in spring in 1985–2011. We investigated how temporal shifts in ringed seal reproduction affect kill composition and the intraspecific vulnerabilities of ringed seals to polar bear predation.

**Principal Findings:**

Polar bears primarily preyed on ringed seals (90.2%) while bearded seals (*Erignathus barbatus*) only comprised 9.8% of the kills, but 33% of the biomass. Adults comprised 43.6% (150/344) of the ringed seals killed, while their pups comprised 38.4% (132/344). Juvenile ringed seals were killed at the lowest proportion, comprising 18.0% (62/344) of the ringed seal kills. The proportion of ringed seal pups was highest between 2007–2011, in association with high ringed seal productivity. Half of the adult ringed seal kills were ≥21 years (60/121), and kill rates of adults increased following the peak of parturition. Determination of sex from DNA revealed that polar bears killed adult male and adult female ringed seals equally (0.50, *n* = 78). The number of hunting attempts at ringed seal subnivean lair sites was positively correlated with the number of pup kills (*r^2^* = 0.30, *P* = 0.04), but was not correlated with the number of adult kills (*P* = 0.37).

**Conclusions/Significance:**

Results are consistent with decadal trends in ringed seal productivity, with low numbers of pups killed by polar bears in spring in years of low pup productivity, and conversely when pup productivity was high. Vulnerability of adult ringed seals to predation increased in relation to reproductive activities and age, but not gender.

## Introduction

Reproduction can incur considerable survival tradeoffs, including increased risk of predation. Mating competition, copulation, and parental care can increase detection of prey by predators, as well as energetically exhaust prey, reducing vigilance against predation [1], [2], [3]. Sexually dimorphic traits associated with mating success can also increase intraspecific vulnerability to predation, as many predators exhibit sex-selective prey choice (e.g. [4], [5], [6]). Synchrony in the parturition of prey swamps predators with an abundance of physically weaker and less experienced prey [7]. As a result, predators are responsive to prey reproductive cycles and the associated vulnerability of reproductive adults and their young.

Polar bears (*Ursus maritimus*) are obligate carnivores, and enter a period of hyperphagia during spring, facilitated by the reproduction and mating cycle of their prey [8], [9], [10]. Polar bears of the Beaufort Sea primarily feed on ringed seals (*Pusa hispida*), and occasionally bearded seals (*Erignathus barbatus*), both of which reproduce and mate between late March and late May [8], [11], [12], [13], [14]. Success rates for polar bears hunting in winter are thought to be low [15], and evidence suggests polar bears are less active during this period [16], [17]. As a result, most polar bears are at or near their minimum body mass for the year in March [9]. Hyperphagic behaviour in spring allows polar bears to increase their mass before the onset of the open water season [9], when reduced prey availability can result in the onset of a fasting physiological state similar to hibernation in other bear species [18], [19], [20], [21].

Previous studies of seals killed in the spring by polar bears suggest the proximate mechanism for prey access for polar bear hyperphagia is the synchronous birth of ringed seal pups, whom are vulnerable to surface predators [8], [10], [11], [22]. In shorefast sea ice areas, polar bears can be significant predators of ringed seal pups, killing up to 44% of the pup production in an area [10]. As such, the proportion of seal pups killed by polar bears in spring is sensitive to seal natality. Although sample sizes were limited, surveys of ringed seals killed by polar bears between 1971–1975 in the Beaufort Sea and Amundsen Gulf showed a marked decrease in the proportion of pup kills in years with lower ringed seal natality, a conclusion that was supported by the simultaneous occurrence of reduced ovulation rates [11], [23]. Lower ringed seal ovulation rates were documented again in 1985–1987 at Sachs Harbour [24] and in 2003–2006, at Ulukhaktok [25]. However, the affect of lower ringed seal natality on polar bear predation and hyperphagia during these periods is unknown.

In addition to the increase of vulnerable pups, adult ringed seals may be at a heightened risk of predation in spring relative to other times of the year. During the open water season ringed seals are pelagic, and polar bears rarely catch seals without having access to them from sea ice [11], [26] (but see [27]). As maximal sea ice extent in the Arctic is reached in March [28], ringed seals in early spring are confined to using self-maintained breathing holes, limiting surfacing options. Due to reproductive activities and mating, ringed seal adults spend nearly 50% of their time out of the water in April and May, much higher than previous months [29]. Limited surfacing areas and increased time spent near or on the ice platform by adult ringed seals may increase the hunting success rate for polar bears. Adult female ringed seals birth and nurse pups in subnivean lairs [30], [31], and it has been hypothesized that killing ringed seal pups at the lair may provide polar bears a secondary opportunity of capturing the adult female [8], [12], [22], [32], although the success of this tactic is unknown. Diving profiles of adult male ringed seals during the breeding season indicate they spend more time near the surface to mark and guard shared breathing holes [29], [33]. Scent marking by adult males is a conspicuous form of mate signaling [34], [35], and likely increases the chances of detection by polar bears. It has been suggested, however, that the odour of breeding male ringed seals is strong enough to confer an anti-predation benefit from polar bears [10], [22], [31], although this has only been examined with hunting attempts on subnivean liars, not seal kills.

The objectives of this study were to: quantify the composition of species, age, and gender of seals killed by polar bears in spring in the Beaufort Sea; investigate how temporal shifts in ringed seal natality affect kill composition; and, test hypotheses on the intraspecific vulnerabilities of ringed seals to predation. If ringed seal natality rates affect the overall composition of kills by polar bears in a particular year, the proportion of ringed seal pups killed should be greater in years with high rates of ringed seal ovulation. In addition, if reproductive activities increase vulnerability to predation, more adult seals should be killed following the peak of parturition. Furthermore, if the strong and apparently unpleasant smell of adult male ringed seals in spring reduces their attractiveness to predators, it might be predicted that males would be killed less frequently than adult females. Finally, if ringed seal vulnerability increases because of exposure to predation at subnivean lairs, then a positive correlation should exist between the number of observed hunting attempts on lairs and both pup and adult kill rates. Observations of both pup and adult female ringed seal kills should occur at the same location, if polar bears are able to catch a ringed seal mother after killing her pup.

## Materials and Methods

**Figure 1:**
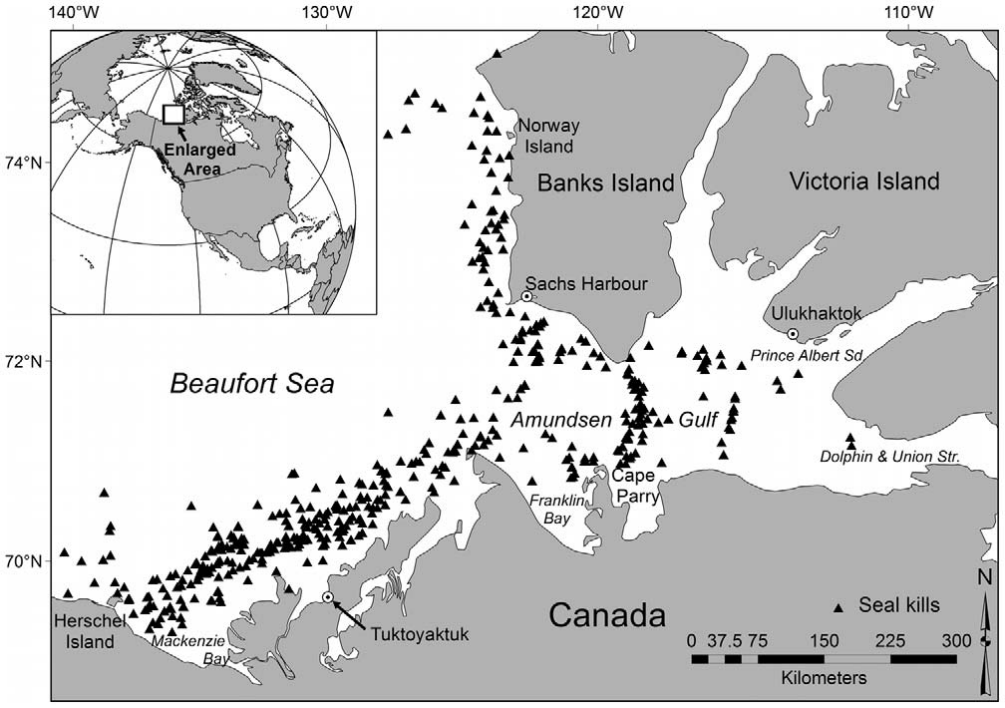
Study area in which seals killed by polar bears (▴) were observed between 1985–2011.

Observations of hunts (digs) and seals killed by polar bears were collected between early-April and late-May (range April 3 – May 28) in 1985–1987, 1992–1994, 2000, and 2003–2011. Observations were gathered opportunistically during polar bear inventory and ecology research. The study area was the eastern Beaufort Sea east of 141° W and south of 75° N, and the Amundsen Gulf (Figure 1). Helicopter flights originated from Tuktoyaktuk, Sachs Harbour, Ulukhaktok, Cape Parry, and Norway Island and were limited to within 150 km of the coast. Search effort included surveying active ice near leads and stable shorefast ice areas [36].

Digs and kills were identified from the helicopter during tracking of polar bears. Digs were categorized as snowdrifts along pressure ridges that were dug into by polar bears. Kill sites were confirmed by the presence of blood, carcass, remains that have been scavenged, or carcasses that were actively being fed upon by polar bears. Due to logistic restrictions, it was not possible to land and investigate all kill sites, so some were noted only from the air. When possible, sites with remains present were investigated and tissue, jaw, and claw samples from kills were collected. Species, age class, and gender were also noted when possible. In some cases where only blood remained, if the amount of blood was minimal, and it was found near a pressure ridge with a dig nearby, it was assumed to represent the kill of a ringed seal pup [26]. Observations of white lanugo at the kill site also helped confirm ringed seal pup kills when few remains were present. Seal kills by Arctic fox (*Alopex lagopus*) were excluded based on the presence of fox tracks and the absence of polar bear tracks.

Tooth histology and claw samples were used to augment seal kill age class observations. Canine seal teeth were extracted from recovered jaws, decalcified, and aged to the year following Stewart *et al.* [37]. Claw samples were employed only to categorize a kill as either pup (0+ years), juvenile (1–6 years) or adult (≥7 years), because claw wear precludes accurately aging beyond 10 years [38].

DNA analysis was used to confirm species and identify gender of seal kills. Seal samples were stored at −20°C and included all observation years except 1985–1987. Extracted DNA of 147 seal kill samples were analyzed by Wildlife Genetics International (Nelson, British Columbia). DNA profiles for both species and gender yielded clear results (D. Paetkau, personal comm.). Results of laboratory age and species classifications were compared with field notes to test the strength of *in situ* records. This test assessed the confidence in the use of field observations when laboratory analysis was not available for confirmation.

Seal kill observations were pooled over all years (1985–2011), and pooled by time periods with data from Stirling and Archibald [11], associated with high (1971–1973; 2007–2011) or low (1974–1975; 1985–1987; 2003–2006) ringed seal ovulation rates. Differences in the proportion of kills between age classes within species, and between years within age classes, were tested for statistical significance using a Pearson Chi-square. The Marascuilo procedure was used for *post-hoc* analysis, as it allows for the comparison of proportion data of several populations simultaneously, using a Chi-square statistic [39]. Given that species was not identified in 31% of the 650 seal kill observations, and age-class was not identified in 58% of the observations, multiple imputation methods were considered [40]. However, Little's MCAR test was not significant (*χ^2^* ^= ^1.35, *df* = 1, *P* = 0.25), and pooled input values did not result in a significantly different estimate of the proportions of species or age-class; indicating little bias in using only complete data. Therefore, all proportions are presented using complete data only. Identified kills were also converted to total biomass for comparison following Derocher *et al.* [26]. Mean kill and dig observations per flight day were compared between 1985–1987 and 2003–2006 using *t*-tests, adjusted to seasonal day (April 11-May 10).

Seal kill observations were pooled into five-day intervals (*n* = 8) to test for the presence of within season trends. A Kruskal-Wallis test was used to analyze whether the distribution of kill observations was equal across time intervals. *Post-hoc* pairwise comparisons using Kruskal-Wallis ranks tested whether kill rates at individual time intervals differed.

Linear regression was employed to test for a relationship between the number of digs (independent) and the number of adult or pup ringed seals killed (dependent) each year. The correlation used data from the years 1985–1987, and 2003–2006, because these years had high sampling intensity (range 26–47 days), and dig observations were consistently recorded. Data were pooled (*n* = 14) into observations that occurred early in the sampling season (≤ April 25) and late (≥ April 26). Regression was performed on Box Cox transformed data ([41]; λ = 0.5), to meet the assumptions of normality (Shapiro-Wilk, *P*>0.05). All statistical tests were conducted in SPSS 18.0 (IBM, Chicago, Illinois), and 95% confidence intervals are reported with all means, unless otherwise stated. For all significance tests, alpha was set to 0.05.

## Results

Between 1985–2011, 369 helicopter flight days were flown over the Beaufort Sea, during which 650 kills and 1396 digs were recorded. Sampling effort between years varied with 72.9% of total flight days recorded in 1985–1987 and 2003–2006, accounting for 77.2% of the kills and 80.9% of digs.

**Figure 2:**
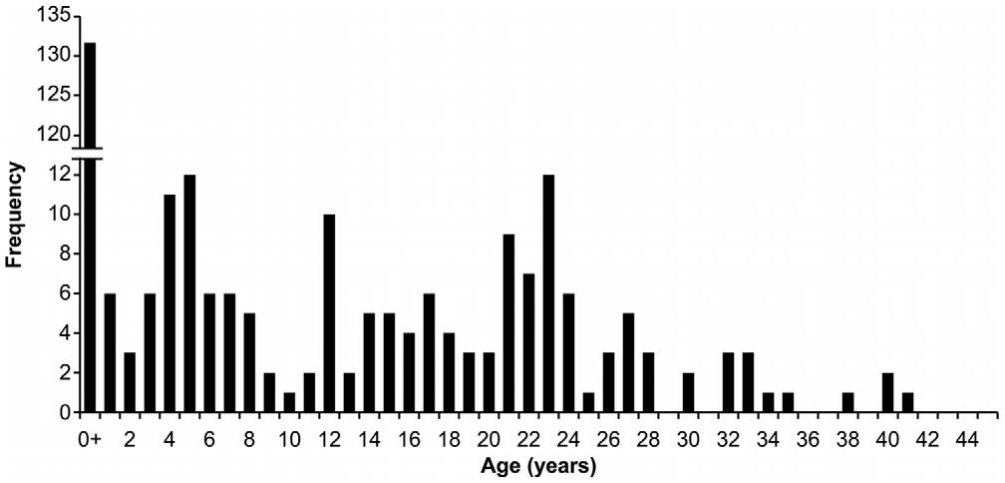
Age structure of ringed seals killed by polar bears in spring between 1985–2011, as determined by tooth histology and field observation (pup age class 0+).

Of the 650 kills, species was undetermined for 200. Ringed seals accounted for 90.2% (406/450) of kills of known species, while bearded seals accounted for the remaining 9.8% (44/450). DNA analysis agreed with the field assessment of species classification in 94.1% of the cases where both were recorded (*n* = 102). Of 450 samples from known species, age class was determined for 344 ringed seals and 32 bearded seals. For ringed seals, 38.4% (132/344) were pups, 18.0% (62/344) were juveniles and 43.6% (150/344) were adults. Overall, ringed seal pup and adult age classes were killed at a higher proportion than juveniles (*P*<0.001, *n* = 344). Of the ringed seal adults, 49.6% were ≥21 years of age (60/121), with the oldest being a 41 year-old female from Dolphin and Union Strait (Figure 2). The eight oldest ringed seals aged by tooth histology and identified by gender were all female. The oldest male was 30 years of age. Mean age for killed adult male ringed seals was 20.7±1.9 years and 22.6±3.4 years for adult females and did not differ by sex (*t* = 1.11, *df = *62, *P* = 0.27). For bearded seals, 25.0% (8/32) were pups, 40.6% (13/32) were juveniles and 34.4% (11/32) were adults. Proportions of bearded seal kills did not differ by age class (*P*≥0.40), although the number of known age class samples was small (*n* = 32). When identified kills were converted to biomass, ringed seals contributed 67% of the overall prey biomass, while bearded seals contributed 33%. Tooth histology classification of seal ages by adult, juvenile, and pup, agreed with field assessment in 87.9% of the cases where both were reported (*n* = 33). DNA analysis of gender of ringed seal adult kills determined the sex ratio as 0.50 (*n* = 78).

**Figure 3:**
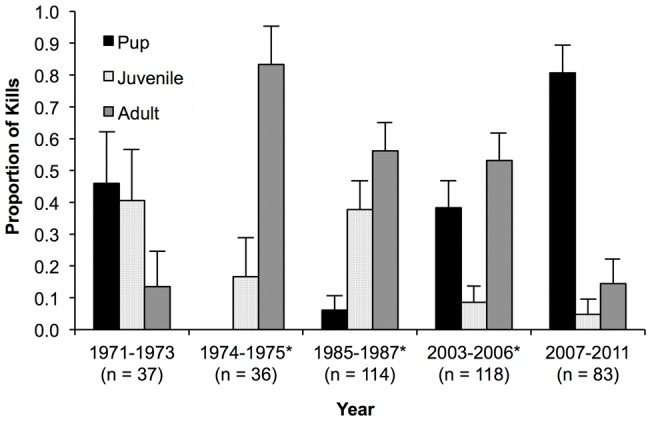
Proportions of ringed seals killed by polar bears in the eastern Beaufort Sea between 1971–2011, categorized by pup (0+ years), juvenile (1–6 years) and adult (≥7 years) age classes (95% CI shown). Data from 1971–1975 reproduced with permission from Stirling and Archibald [11]. *Years with lower ringed seal reproduction as recorded at Sachs Harbour and Ulukhaktok [24], [25], [63].

Age class proportions of ringed seal kills from this study along with Stirling and Archibald [11], were not distributed evenly between time periods associated with high and low ringed seal ovulation rates (*χ^2^* ^= ^176.8, *df = *8, *P*<0.001, Figure 3). Ringed seal pups were killed at the highest proportion from 2007–2011 as compared to any other time period (*P*<0.01). Proportions of adult ringed seal kills were lowest in 1971–1973 and 2007–2011 compared to the other periods (*P*<0.001). In 1985–1987, 2.20 kills/flight day were observed, which was not different than the 1.84 kills/flight day in 2003–2006 (*t* = 1.07, *df* = 228, *P* = 0.28). In 2003–2006, 5.16 digs/flight day were observed and was significantly higher than the 2.36 digs/flight day in 1985–1987 (*t* = −2.87, *df* = 228, *P*<0.01). Mean estimated age of adult ringed seals killed increased from 17.9±1.8 years between 1985–1994 to 21.6±2.3 years between 2000–2011 (*t* = 2.51, *df* = 113, *P* = 0.01).

**Figure 4:**
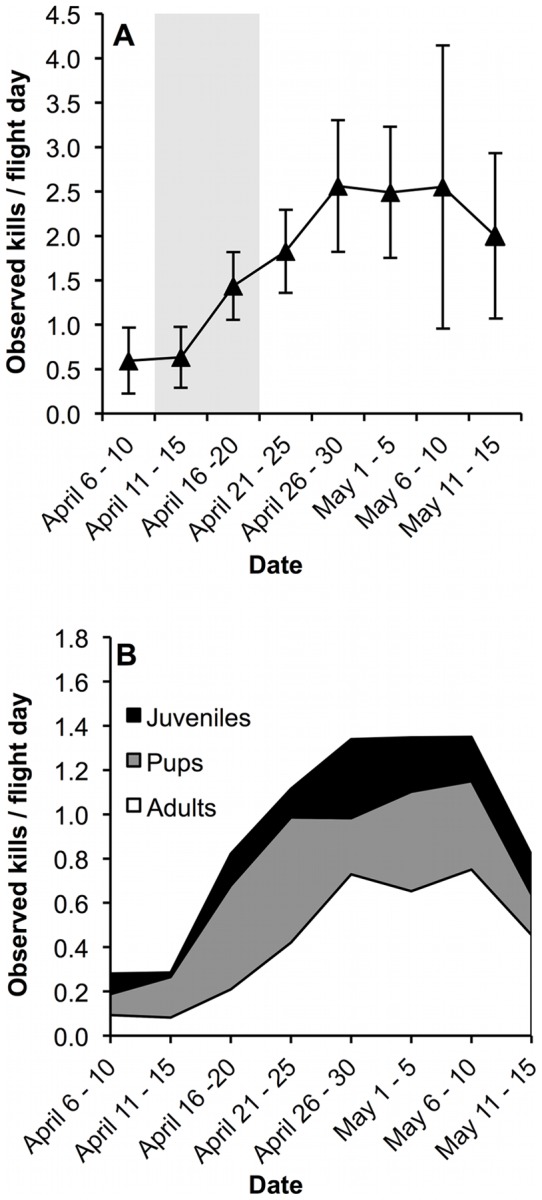
Mean daily number of kills observed per five-day period in the eastern Beaufort Sea between 1985–2011. 4a: Mean daily number of all kills observed (± SE); error represents combined daily and annual variation in observations. Grey shading indicates peak ringed seal whelping in the Beaufort Sea [12]. 4b: Mean number of ringed seal pup (0+ years), juvenile (1–6 years) and adult (≥7 years) kills observed.

Abundances of seal kill observations were not distributed evenly over the season (*H* = 47.5, *df* = 7, *P*<0.001, Figure 4a). *Post-hoc* analysis revealed kill observations were significantly higher from April 21 – May 5 compared to April 6 – 15 (*P<*0.01, Figure 4a). Pup and juvenile ringed seal kill observations per day (*n* = 40) did not differ over time (*H*_pup_ = 12.5, *P*_pup_ = 0.09; *H*_juv_ = 8.6, *P*_juv_ = 0.29; *df* = 7, Figure 4b). Observations of adult ringed seal kills per day varied (*H* = 17.8, *df* = 7, *P* = 0.01), as a *post-hoc* examination revealed that the number of kills observed was higher April 26 – 30 compared to April 6 – 15 (*P*<0.05, Figure 4b). Temporal correlation between daily observation rates of total kills and adult ringed seal kills was evident (Spearman rank correlation, *r_s_* = 0.69, *P*<0.001, *n* = 40).

**Figure 5:**
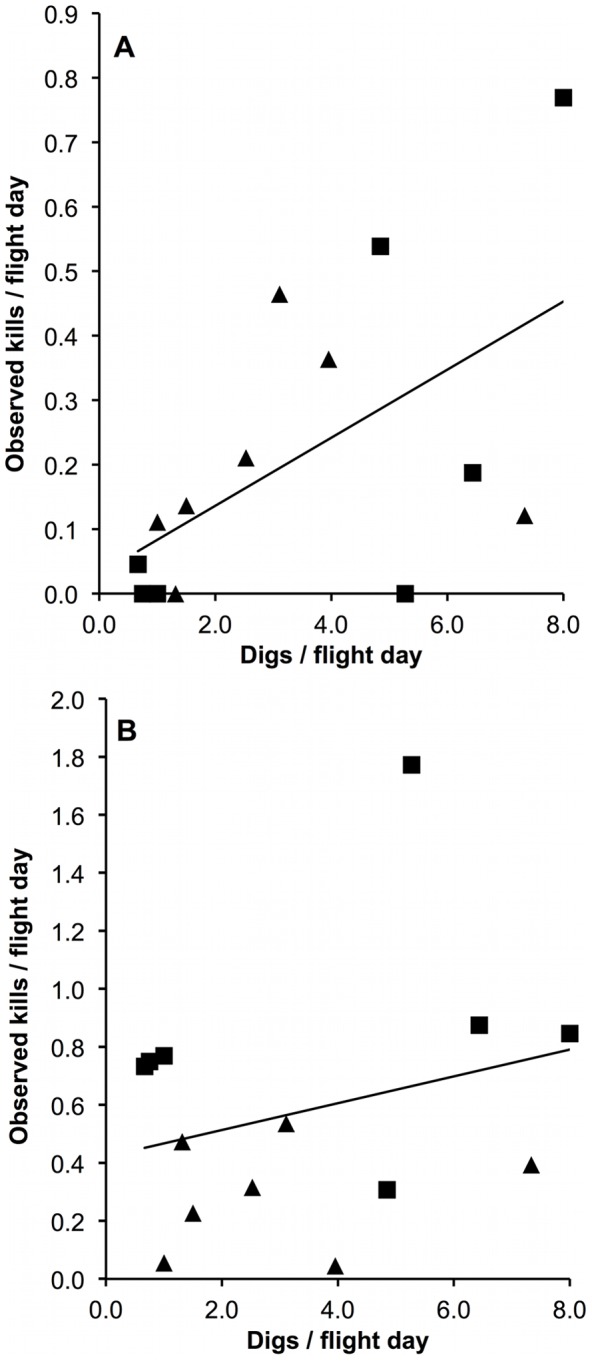
Relationship between attempted hunts on subnivean ringed seal lairs (digs) observed per flight day and ringed seal kills. 5a: pups (0+ years, *r^2^* = 0.30, *P* = 0.04). 5b: adults (≥7 years, *P* = 0.37). Observations were pooled (*n = *14) into early (▴, ≤ April 25) and late season (▪, ≥ April 26). Data shown untransformed; see text.

There was a positive correlation between the number of digs observed and the number of ringed seal pup kills (*r^2^* = 0.30, *df = *12, *P* = 0.04, Figure 5a) but no correlation between the number of digs observed and the number of adult ringed seal kills (*r^2^* = 0.07, *df = *12, *P* = 0.37, Figure 5b). Additionally, there were no observations of a pup and adult ringed seal killed at the same location. The closest proximity of a pup and adult kill was observed on April 29, 2009, when an adult female kill was found 1.76 km from a pup kill.

## Discussion

Extrapolating foraging behaviour of polar bears from opportunistic observations is subject to potential bias. Search effort in our study was not systematic, as kills were found while following polar bear tracks. This resulted in a high representation of the floe edge and moving ice habitats that polar bears show a preference for [36], while underrepresenting other habitats, potentially resulting in some bias in kill composition. However, because the objective of the research was to locate as many polar bears as possible, it is likely that the sampling reflected areas where polar bear foraging was successful, reducing the possibility of missing substantial numbers of kills in other habitats. Searching for kills was also dependent on the spatial scope of polar bear ecology and inventory research. Most research flights were flown between the Tuktoyaktuk Peninsula and Herschel Island, or on the western and southern coasts of Banks Island and the western entrance to Amundsen Gulf, with less time spent further east in the Amundsen Gulf. Additionally, between 2007–2011, research flights were based only from Tuktoyaktuk, resulting in search effort restricted to the southern Beaufort Sea. As depth contours and sea ice conditions vary throughout the eastern Beaufort Sea and Amundsen Gulf, extrapolating from one area may not fully depict foraging behaviour for polar bears across the study region. Nonetheless, we believe our samples are broadly representative of the seals killed.

Species composition in the diet of polar bears of the Beaufort Sea was similar to that reported in past studies [11], [14]. Polar bears primarily preyed upon ringed seals, with only small numbers of bearded seals being predated which, in part at least, reflects the relative abundance of the two species in the study area [42]. However, because subadult and adult bearded seals are substantially larger than ringed seals, it appears that the majority are killed by adult male polar bears, though carcasses may be scavenged by younger animals [14], [43], [44]. Although the numeric contribution of bearded seals to the kill composition is low, bearded seals contributed approximately one-third of the kill biomass. However, caution is warranted in interpreting the biomass composition to be anything but a rough estimate. The estimate of dietary contribution of bearded seals in this study is higher than from previous estimates using fatty acid analysis [14]. As the estimation technique pools juveniles and adults into the same weight class for each species [26], the calculation may have upwardly biased bearded seal contribution. Nonetheless, the finding supports polar bear dietary studies in other regions, which have found bearded seals to be an important contributor to the overall biomass intake [22], [26].

Age class composition of ringed seal kills varied temporally, in general association with years of low and high ringed seal ovulation rates. Ringed seal pups were killed at the highest proportion between 2007–2011, when ringed seal ovulation rates were over 90% [25]. The result suggests that when ringed seal recruitment is high, polar bears kill mostly ringed seal pups in spring. Ringed seal juveniles were killed half as frequently as adults between 1971–2011, which was unexpected given that polar bears focus on younger age classes during predation [8], [11], [15], [22]. However, this result may also reflect that the majority of kills were observed in years with lower ringed seal productivity. Juvenile ringed seals were observed to decrease in Inuit open water catches for two to three years immediately following low ringed seal natality [12], [24]. These results support the suggestion that the decadal cycle of ringed seal productivity affects the kill composition of polar bears in the spring [23], [45].

Observations of juvenile ringed seal kills were lower in 2003–2011 than in 1985–1987. The mechanism for the decline is not well understood. Juvenile ringed seals in the study area have been observed to be in worsening body condition over the past two decades [25]. Coupled with an increase in the average age of adult ringed seal kills, decreases in juvenile representation in the kill composition could be symptomatic of a declining population. However, our understanding of juvenile ringed seal behaviour and distribution are still inadequate. Juvenile ringed seals do not restrict themselves to a territory, and will spatially segregate themselves from adults during early spring to take advantage of high quality foraging areas [46]. This may translate into an unpredictable source of prey for polar bears, and disentangling the predator-prey effects from possible population effects is difficult. As such, it is unwarranted to speculate further on the causes of the observed trends.

Seasonal analysis indicated an increase in the rate of observations of ringed seal adult kills and total kills after the peak ringed seal whelping date. A temporal correlation between these two trends suggests ringed seal adult kills may have driven the increase in total observed kill rates. There are two nonexclusive hypotheses for the increased kill rates of adult seals following whelping. First, reproductive behaviour may increase predation risk for adults. Adult female ringed seals are income breeders [47], [48], and have a spatially restricted foraging pattern while nursing [29], [49]. Territorial behaviour in adult male ringed seals peaks post-whelping and less dominant males are excluded from prime-breeding habitat [12], [50]. Additionally, both male and female ringed seals spend an increasing amount of time out of the water during reproduction and mating [29]. The restricted spatial ranges of adults and repeated use of surfacing areas may increase the likelihood of predatory success for a sit-and-wait predator such as the polar bear.

Second, approximately half of the adults killed were ≥21 years, indicating a potential age related mechanism of vulnerability in adults. Although ringed seal life expectancy can range up to 45 years [51], the proportion of the adult population over 20 years old rarely exceeds 30% in catch statistics [12], [51], [52]. Using smoothed age-frequency estimates from Smith [12], ringed seal adults 21 years and older only compose ca. 15% of the adult age class. A high kill composition of pups and older seals supports the controversial theory that as a predator, polar bears may be killing the old and the weak in the prey population [53], [54]. For ambush predators, prey selection is largely limited to what avails itself, and therefore dependent on the behaviour of the prey. In years of high ringed seal ovulation, polar bears have access to a large number of vulnerable pups. In low ovulation years, polar bears diets include a higher proportion of older adult ringed seals, whose potentially more limited mobility (e.g. [55]) may increase their vulnerability.

The finding that adult male and adult female ringed seals were killed in similar proportion is contrary to the prediction that polar bears avoid adult males during spring. Previous studies had noted that hunting polar bears ignored ringed seal subnivean lairs with a strong rutting male scent [10], [22], [31]. Explanations for this avoidance included: the meat of rutting male ringed seals is unpalatable to polar bears [10], [22], [56]; breeding odour serves to confuse the olfactory senses of polar bears during hunting [22]; or adult males in subnivean lairs are more difficult to catch for polar bears than younger age classes [10], [31]. Results from this study suggested adult male ringed seals comprised a significant portion of the polar bear diet in spring, and therefore the only hypothesis supported by this study is the last: adult males may be more difficult to catch in stable ice subnivean lairs.

The number of observed attempted hunts on subnivean lairs (digs) was positively correlated with the number of pup kills, but not correlated with the number of adult kills. This observation is consistent with evidence that attacks on subnivean lairs in stable ice are predominately aimed at ringed seal pups [10], [22], [56]. However, there is a hypothesis that in cases where a pup kill provides limited energetic return, polar bears may attempt to exploit the mother-pup bond, and capture the adult female [8], [12], [22], [32]. Yet, during our study we found no support for such a hunting strategy.

Stirling and McEwan [8], reported that some of the newborn ringed seal pups killed at lairs are unconsumed, and given pups low energetic value and fat content during nursing, they suggested polar bears may have been hunting the adult females. Given dig success rates can be less than 10% [11], and polar bears are inefficient walkers [57], searching and digging for ringed seal pups alone may not result in a net energy gain. Due to the inability to screen out scavenging of kills by other predators, relative consumption rates were not examined in our study. However, despite pups' daily gain in fatty tissue [58], the number of pup kills we observed per day between mid-April and early May remained relatively constant. Two hypotheses could support these observations. First, the daily increase in the mass of ringed seal pups provides progressively greater thermal insulation, and pups spend more time in the water column as the nursing period progresses [59]. Reduced vulnerability to predation may counteract increased hunting effort by polar bears, explaining the relatively constant kill rate within season. Second, preying on ringed seal pups may be part of a greater overall strategy of polar bear females protecting cubs (<1 year old) in spring, and lower energetic gains are a consequence of their habitat selection. Habitat selection studies in the Beaufort Sea suggest that female polar bears with cubs select stable, shorefast ice habitat with subnivean lairs, segregating themselves from the rest of the polar bear population [36]. It is hypothesized that females with cubs avoid adult males [36], [60] due to risk of infanticide and being killed themselves [61], [62]. Adult females with cubs may trade reduced energetic input for protection of young during this period, which could contribute to the high proportion of ringed seal pup kills, despite the pups' limited energetic value.
